# Enchondroma versus Low-Grade Chondrosarcoma in Appendicular Skeleton: Clinical and Radiological Criteria

**DOI:** 10.1155/2012/437958

**Published:** 2012-04-22

**Authors:** Eugenio M. Ferrer-Santacreu, Eduardo J. Ortiz-Cruz, José Manuel González-López, Elia Pérez Fernández

**Affiliations:** ^1^Unidad de Tumores Musculoesqueléticos, Servicio de Cirugía Ortopédica y Traumatología, Hospital Universitario La Paz, Paseo de la Castellana 261, 28046 Madrid, Spain; ^2^Departamento de Bioestadística, Hospital Universitario La Paz, Paseo de la Castellana 261, 28046 Madrid, Spain

## Abstract

*Objectives*. To determine the validity of clinical and radiological features of enchondroma and low grade chondrosarcoma, and contrast the biopsy results with the clinical diagnosis based on the history and imaging. *Material and Method*. The study included 96 patients with cartilage type lesions suggestive of an enchondroma (E) or an low grade chondrosarcoma (LGC) according to the clinical and imaging data. The hypotheses were contrasted with the biopsy. *Results*. Of the 82 patients studied completely, 56 were considered E (68.29%), 8 as LGC (8.33%) and in 18 (18.75%) were doubtful cases and considered as suspected LGC. Of these, the biopsy showed 4 E (25%), 10 LGC (50%) and 4 were not definitive. On the other hand, of the 56 cases diagnosed as E, 15 were biopsied, 5 of these biopsies turned out to be LGC (33.3%). The 8 cases diagnosed as LGC, were also biopsied and only 4 biopsies (50%) confirmed the initial diagnosis. Features analyzed in the study showed no statistically significant difference. Correlation analysis between the diagnosis issued initially and the biopsy result gave a value of 0.69 (kappa coefficient), which was considered a good correlation. *Conclusion*. Features analyzed did not have any statistical significance. However, there was a good correlation between initial diagnosis and biopsy's result.

## 1. Introduction

Bone tumors diagnosis includes many radiological, epidemiological, pathological, and clinical considerations. Those including patient questioning, physical examination, and radiology will give some hints towards the final diagnosis. But, most of the time, a specimen must be obtained in order to guarantee an accurate confirmation. However, in some cases, it is not easy to decide what kind of malignancy we are treating even with a biopsy. It is in those situations that clinical and radiological data become extremely important when making a decision on treatment strategies. Among cartilage tumors, two entities can be misleading for the pathologist when trying to reach a correct diagnosis: enchondroma (E), a benign tumor, and low grade chondrosarcoma (LGC), which is a low-aggressivity malignancy. This can be a problem when deciding treatment because E only requires regular followup but LGC needs surgical treatment. The goals of the present study are first, to determine the existence of statistical significance between clinical and radiological criteria (obtained from physical examination, plain radiographs, computerized tomography (CT), magnetic resonance (MRI), bone scan with Tc99) and biopsy results; secondly, to find the correlation degree between surgeon's initial impression considering only clinical and radiological data and pathologist's diagnosis on the biopsy. Clinical, radiological, and histological data of both E and LCG are summarized in Table 1.

## 2. Material and Methods

We have performed a prospective study in which 96 patients treated by the authors in our institution. On that purpose, databases from the institution and from the Pathology Department of the biggest center were used. All studied patients showed a cartilage-type lesion suggesting E or LGC on plain radiographs. Further imaging (CT, MRI, and Tc99 bone scan) was performed making an initial impression by the orthopaedic surgeons, which was compared to the final diagnosis (E or LGC) made by the pathologist. Exclusion criteria were established as follows: patients less than 18 years old, multiple enchondromatosis or multiple osteochondromatosis, chondrosarcomas on previous osteochondromas, cartilage lesions in hands or axial skeleton, and grade II and III chondrosarcomas according to Evans' classification.

For each patient, a form was fulfilled with clinical and radiological information, including personal data, treating center, physical examination, symptoms (focusing on pain), and followup. Age was divided in two categories, under 35 and 35 or older. In plain radiographs, tumor site, side, and affected bone segment., size and calcifications (changes over time) were recorded. In CT, size, calcifications (presence and changes over time), endosteal scalloping, and soft tissue mass (STM) were registered. In MRI, size, endosteal scalloping and STM were also recorded. Concerning Tc99 bone scan, lesions were classified according to the presence of radionuclide uptake on whole-body image. The degree of uptake was compared to anterior iliac crest activity (similar, lower, or higher than iliac crest physiological uptake) focusing on anterosuperior iliac crest, as recorded by nuclear medicine specialists in their reports. In each case, an initial diagnosis (E or LGC) was made according to the clinical and radiological data. Finally, a record of the pathology (type of procedure and result) was made after pathologist's reports, confirming or not surgeon's initial judgment. Those cases considered initially as E were classified in the E group if no changes in clinical and radiological data were recorded after 5 years, even if the biopsy was not performed.

Statistical analysis was performed by a member of the Statistics Department. Microsoft Excel and SPSS software were employed to obtain *P* values and correlation between initial diagnosis and histological results (kappa coefficient).

Documentation and references were gathered by means of PubMed and Ovid search tools using “enchondroma versus low-grade chondrosarcoma” and “chondral tumors diagnosis” as keywords. Articles older than 20 years were discarded, except those considered classic keystones on the matter.

## 3. Results

As a whole, data from 96 patients have been analyzed, 22 men and 73 women. Most of the patients showing an E after the biopsy were women (92.9%), so happened with LGC (88.9%); hence, sex was not an statistically significant factor in our study. Mean age was 50.82 years, and 84.4% of the patients were more than 35 years old. Distribution of E and LGC between the two age groups (younger than 35 and 35 and older) was almost equal (50%/50% for E and 41.4%/58.6% for LGC). No relationship was found concerning patient's age. Most common location was the femur (52.1% of the cases) with no clear predominance of any side (48.3% in right side and 58.5% in the left side). Metaphysis was the most common segment affected: 28.1% proximal, especially in the humerus, and 25.8% distal, especially in the femur. None of these features was statistically related to E or LGC, comparing them to the biopsies. Concerning the size of the mass, 71.2% were smaller than 5 cm. All biopsies showing E were less than 5 cm. But only 50% of those showing LGC were smaller than 5 cm. Even with these differences, no statistical significance was found between size and final diagnosis (*P* > 0.05 in Fisher's exact test). According to registries in our study 44.7% of the cases were incidental findings and 55.3% were found after a patient's consultation because of pain or other symptoms as discomfort. None of these situations showed any statistical significance with the final diagnosis. 40% of the patients complaining of pain at the first visit were finally diagnosed as E and 60% as LGC. Only 14.3% of the patients undergoing a biopsy had no pain at all. Pain was not an statistically significant factor in our study. 69.2% of the patients belonging to the E group and 61.2% of the LGC group complained of mechanical pain; so this feature was neither considered as statistically significant. In physical examination, all patients from E group and 94.4% from the LGC group had tenderness in the surrounding area. This fact was not statistically significant.

**Figure 1:**
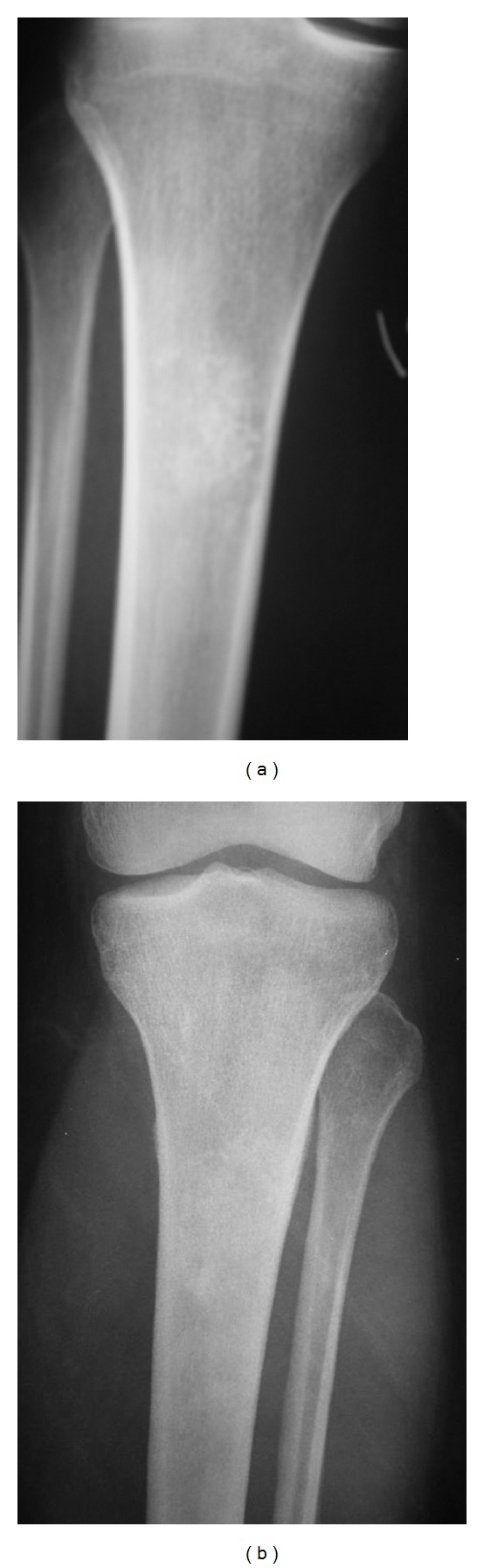
Cartilage lesion in proximal tibia in which calcifications changed over time (A1994 image B 2000 image).

All lesions included in our study showed some degree of calcification. In two cases, these calcifications suffered lysis within a previously calcified area over time (Figures 1(a) and 1(b)). In 60% of the E patients, 70.6% of the LGC cortical resorption was seen on CT. Most of the time, it affected one third of the cortical or even less (30% of E and 64.7% of the LGC). No soft tissue masses (STMs) were found on CT in our study. On MRI, 25% of the E and 52.4% of the LGC showed endosteal scalloping. As on CT, most of the cases affected one third or less of the cortical, 16.7% of the E and 42.9% of the LGC. There was one case showing STM (only detected on MRI) in a patient who was finally diagnosed as LGC. None of these results on CT and MRI was statistically significant in our study even if there were some differences. Concerning bone scan, all patients showed active lesions for both diagnosis. 60% of the E had the uptake level similar to anterosuperior iliac crest, 30% showed more uptake, and 10% less uptake. Among LGC patients, 52.9% had the same uptake, 41.2% had more uptake, and 5.9% had less uptake. No statistical differences were found in these data.

Concerning the type of procedure performed in order to obtain specimens in each patient, the distribution is as follows: 8 percutaneous core-needle biopsies, 8 incisional biopsies, and 22 excisional procedures (intralesional wide resection with adjuvant therapy). In 3 cases, one inconclusive core-needle biopsy had to be confirmed by an incisional procedure. There were no statistical differences analyzing the degree of correlation (kappa coefficient) according to the type of procedure performed. A reason for this is the small amount of patients in each category except for incisional and excisional procedures.

Initially, 56 out of 82 patients were considered as E (68%), 8 as LGC (8%), and in 18 cases (18%) it was impossible to obtain a clear judgment. They were included in the group considered as clinical and radiological suspicion of LGC. All of them underwent a biopsy. Of these, 4 turned out to be E (25%), 10 were diagnosed as LGC (50%), and the other 4 were inconclusive pathologies (in case of doubt they were treated as LGC). On the other hand, 15 out of 56 cases judged initially as E and 15 underwent a biopsy. The reason why we obtained a specimen from a lesion initially considered as E was changes on the physical examination (pain or loss of function) or imaging (calcification lysis or endosteal scalloping). Five of them were finally classified as LGC after pathology's results. The 8 cases judge initially as LGC underwent a biopsy and only 4 confirmed that diagnosis (50%). After all these results, Kappa coefficient showed a value of 0.69, which is considered a moderate degree of correlation between surgeon's initial judgment and pathology's final result.

## 4. Discussion

Differentiation between E and LGC remains a challenge for those physicians involved in musculoskeletal oncology care [1–3]. Even if other diagnosis must be included in the differential (osteomielitis, bone infarction, aneurismal bone cyst) E and LGC are undoubtedly at the top of the list. In addition, there is not much consistent data published on this topic although initial judgment in these patients is critical for correct decision making on diagnosis and treatment.

Orthopaedic surgeons have three treatment options: followup by sequential clinical assessment and radiographic evaluation for patients with cartilage lesions having a benign aspect. Next option is performing a biopsy. There are two kinds of procedures: percutaneous biopsy, performed with a core-needle device, and incisional biopsy. The third option is surgical intervention to obtain the whole specimen; excisional biopsy with adjuvants could be performed. The most appropriate procedure and reconstruction is still a matter of discussion among specialists. Some prefer to perform an “en bloc” resection while others think that intralesional wide resection is a good option considering morbidity and recurrence rate. Intralesional wide resection includes opening a large cortical window to visualize the whole tumor, curettage associated with high-speed burring, and the remaining cavity is supported by some adjuvant therapies as a lavage with a high-pressure pulsatile system, phenolization, or cryosurgery. Finally, the bone defect is packed with PMMA. Making the right choice in each case is a matter of an accurate initial diagnosis with medical history, physical examination, and radiological imaging.

In our search, the most complete article in this matter is the study by Murphey et al. [4] in which the authors analyze clinical and radiological features in 187 patients (92 E and 95 C). Among all features studied, they found that older patients, bigger size, endosteal scalloping involving two-thirds of the cortex or more (considering depth but not width of the scalloping), presence of a soft tissue mass, broken cortices or periosteal reaction in CT or MRI, and uptake higher than iliac crest physiological uptake in bone scan strongly suggested the diagnosis of a LGC. Their comparison is made between enchondromas and chondrosarcomas of all grades; that must be the reason why they find so many relevant differences. in our study, we focused our analysis on E versus LGC trying to define clinical and radiological criteria between these specific two entities, because intermediate and high grade do not represent such a problem in general.

As well, Marco et al. [5] and Weiner [6] described in their reports a general management of benign cartilage tumors and proposed an algorithm to rule out possible malignancies. Following the model of Murphey's study, we have intended to compare our results to theirs.

Clinical presentation of these tumors is a casual finding, when studying the patient for other reasons. In our series bone metaphysis was the most common site, especially proximal humerus and distal femur. Presence of pain has always been related to malignancy or, at least, to a certain degree of aggressivity. In E versus LGC differential diagnosis, it should make us think about LGC. However, it is mandatory to rule out any other pain source in this area before thinking of malignancies, that is, rotator's cuff injuries or calcifications in soft tissue structures of the shoulder, tendinitis, or sprains if we are focusing on distal femur. If the pain does not show a mechanical pattern, this might mean that we are dealing with an active lesion. In our series, pain features were not significantly related to none of both. Murphey et al. [4] found statistical significance between pain and chondrosarcomas.

Brien et al. [7] recommend for a biopsy every epiphyseal E in order to distinguish it from a possible chondroblastoma or a clear cell chondrosarcoma. The differential diagnosis of chondroid lesion in the epiphysis includes chondroblastoma, clear cell chondrosarcoma or even a giant cell tumor, but not an E because epiphyseal E is very rare in long bones. In our series, only two epiphyseal lesions were biopsied, one of them was in distal femur and we did because of pain and size bigger than 5 cm. We thought that it was a LGC and so showed the pathology. It was treated with an intralesional wide excision. The second case was in the fibular head and we decided to make a biopsy because of pain and an increased uptake higher than the anterior iliac crest. Pathology was informed as E but pain persisted so the tumor was resected and the specimen was classified finally as LGC. Same authors suggest prophylactic intralesional wide resection for patients under 35 years old and showing a lesion bigger than 7 cm. considering there is an important risk of malignization all over the years [7]. In our series, we do not have a single case in that particular situation but our general recommendation is to keep annual followup unless any clinical or radiological change occurs.

**Figure 2:**
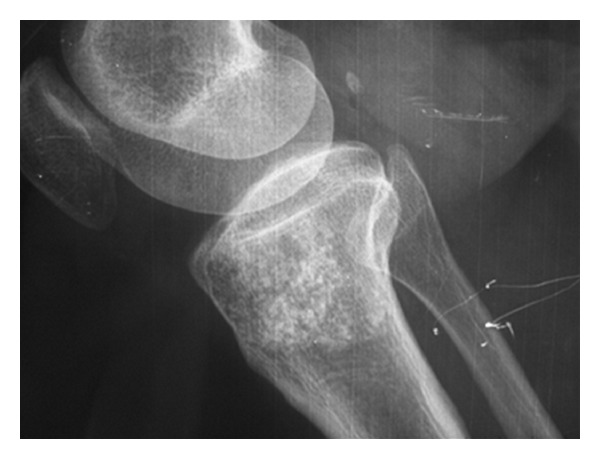
Cartilage lesion in proximal tibia. Malignancy was suspected because of the size, but it turned out to be an enchondroma after biopsy.

**Figure 3:**
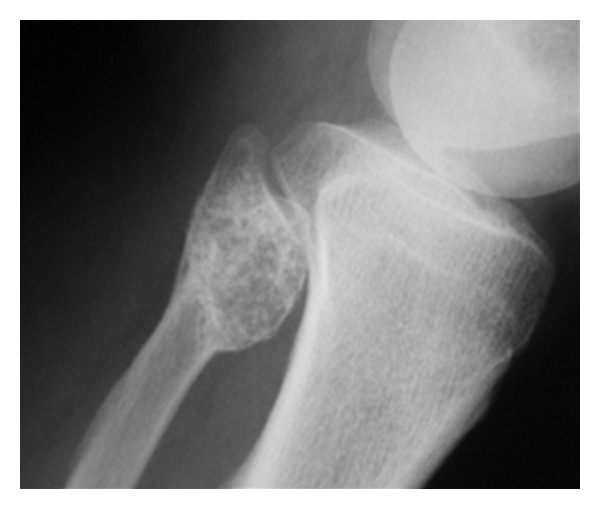
Cartilage lesion occupying epiphysis, metaphysis and diaphysis. Biopsy showed an LGC.

**Figure 4:**
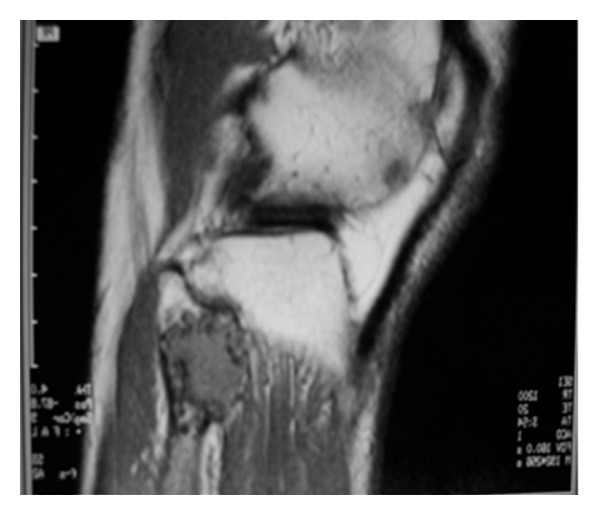
MRI of the same lesion showing cortical damage and soft tissue mass.

On plain radiographs, both tumors have a pop corn-like appearance altogether with arcs and rings pattern (Figures 2 and 3). Although it is the first step for diagnosis and it can show the cartilage, it was not a useful tool to determine the real size, calcification degree, and cortex involvement. It tended to overestimate the measure because it is only a bidimensional image. CT scan and MRI imaging are more accurate when assessing these features (Figure 4). Brien et al. [7] remarks that E has a nodular appearance whereas LGC tends to show a single mass appearance. The reason for this is explained by the fact that E is considered to originate as cartilage remaining of the physeal plate, which are stimulated by some unknown factor. This could be the explanation of the E being multifocal. In Murphey et al's series [4], chondrosarcomas were more common in metaphyseal location, but E was more common in diaphysis. In our study, both tumors were equally common in metaphyseal locations.

In MRI and CT scan, Murphey found statistical significance between chondrosarcomas and common features of malignancy: endosteal scalloping involving more than two-thirds of cortices depth, soft tissue mass, breaking of the cortex and periosteal reaction. In our study, we only assessed how deep was the cortical involvement. We did not found any case showing breaking of the cortex and there was one case of soft tissue mass. Endosteal scalloping affected one third of the cortex in almost all cases in our series (Figure 6).

**Figure 5:**
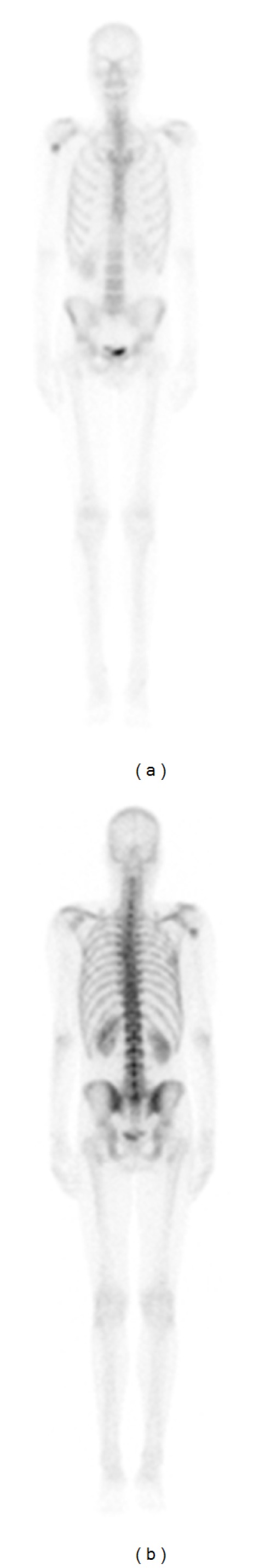
Bone scan showing proximal humerus cartilage mass with an increased uptake higher than anterior iliac crest. It turned out to be an LGC.

**Figure 6:**
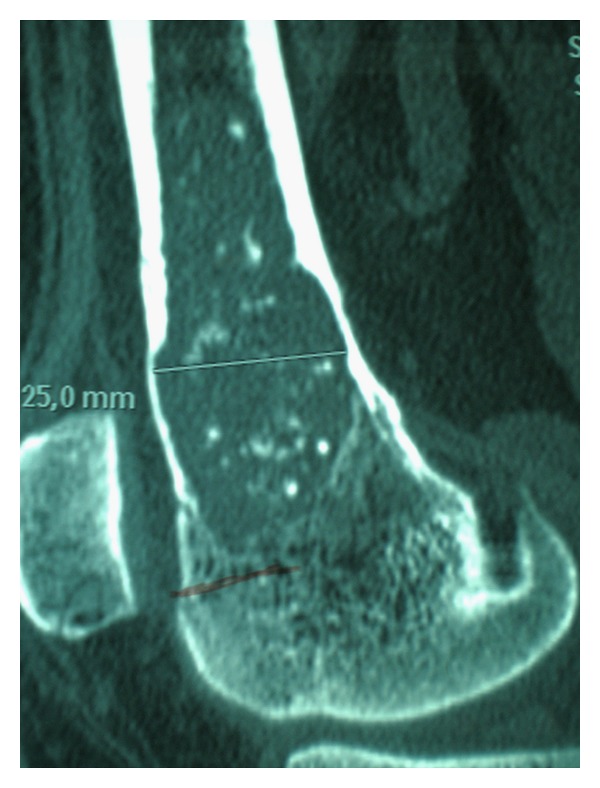
CT scan image showing endosteal scalloping of a distal femur cartilage lesion. Biopsy showed an LGC.

Concerning bone scan imaging, malignancies are expected to have a greater metabolic activity; therefore, LGC should show more uptake than E. Nevertheless, in some circumstances, E might show an increased uptake in bone scan: pathologic fracture, cortical expansion in small bones (not included in our study) or impingement with other anatomical structures next to the tumor site. Murphey could assess these features in 51 patients classifying them in three categories: (1) less uptake than anterior iliac crest (AIC), (2) Same uptake than AIC, (3) Greater uptake than AIC. Authors found that 42 out of 51 patients with chondrosarcoma showed greater uptake than AIC, which was statistically significant (Figure 5). In our study, we did not found any statistical significance.

Correlation between initial judgment made by the senior authors and the pathology result showed a Kappa value of 0.69, considered as moderate/good. Our interpretation is that surgeon's experience is capital in decision making when physical examination, imaging, and pathology result do not help with clear criteria. Senior authors have been focused on orthopaedic oncology for many years. Altogether with radiologists, oncologists, radiation oncologists, pathologists also specialized in bone and soft tissue sarcomas, results in a team work that enables a suitable diagnostic and therapeutic strategy even in difficult cases. In our institution, difficult cases are discussed in multidisciplinary conferences held weekly with all specialists involved in orthopaedic oncology patient care. These practices have improved cooperation between different departments and make therapeutic strategies timing easier for patients and their physicians.

In our understanding, our study showed some weaknesses. Only 82 of 96 patients completed the followup. Of these, only 40 underwent a biopsy. On the other hand, it is a prospective multicenter study, which is a good basis for further data collection and analysis. Furthermore, we only took into consideration type I chondrosarcomas of long bones, excluding axial skeleton in which LGC is more probable; and short bones of hands and feet in which E is nearly always the final diagnosis.

## 5. Conclusions

Benign and low-grade cartilage tumors are not easy to differentiate, and they remain a challenge for physicians in decision making and therapeutic strategies. All specialists involved in orthopaedic oncology can help in differentiating E from LGC for a correct treatment. Our study showed no association between these two entities and clinical and radiological criteria in patient history, physical examination, plain radiographs, CT scan, MRI, and bone scan with Tc99. On the other hand, there was a good correlation between surgeon's initial judgment and pathology results. This enhances the importance of physician's experience and team work when dealing with these cases. More patients should be included to obtain a clear relationship between E and LGC with any of the analyzed features. We strongly recommend multidisciplinary assessment of difficult cases in order to avoid mistakes in decision making which could lead to wrong treatments. Maybe, next step in cartilage lesions differentiation will rely on molecular and genetic tests to clearly determine what kind of tumor we are treating.

##  Disclosure

The authors declare that they have no economical, profesional, or personal conflicts as they have not received any kind of fund or gratification from any public or private institution during the preparation of this paper.

## Figures and Tables

**Table 1:** Compared clinical, radiological and histological features of enchondroma and low grade chondrosarcoma.

	Enchondroma	Low-grade chondrosarcoma
	(i) Younger patients (casual finding in adults)	(i) Patients over 25 years old
History and physical examination	(ii) Seldom painful	(ii) Inflammatory pain
	(iii) Appendicular skeleton almost exclusively (when in phalanx, E almost 100%)	(iii) In axial skeleton, a chondral tumor is always a chondrosarcoma until the opposite is proven
	(iv) In general size <5 cm	(iv) Tends to be bigger than 5 cm

	(i) Normally intramedullary (except for enchondroma protuberans)	(i) Intramedullary
	(ii) No periosteal reaction	(ii) periosteal reaction and associated microfractures
Imaging	(iii) No endosteal scalloping (or minimal)	(iii) Frequent endosteal scalloping
	(iv) No changes during the followup	(iv) Changes over time, such as calcifications disappearance, indicating malignization
	(v) No soft tissue mass	(v) Soft tissue mass

	(i) Typical encasement pattern	(i) Invades Haversian system
	(ii) No endosteal scal loping	(ii) periosteal reaction with endosteal scalloping
Biopsy	(iii) Multinodular aspect	(iii) Ocasional necrotic and haemorraghical focii
	(iv) Surrounded by lamellar bone	(iv) Invades bone marrow
	(v) Does not invade bone marrow	(v) Generally a single mass
